# Transformation of ABT-199 Nanocrystal Suspensions into a Redispersible Drug Product—Impact of Vacuum Drum Drying, Spray Drying and Tableting on Re-Nanodispersibility

**DOI:** 10.3390/pharmaceutics16060782

**Published:** 2024-06-08

**Authors:** Barbara Schönfeld, Julius Sundermann, Benjamin-Luca Keller, Ulrich Westedt, Oliver Heinzerling

**Affiliations:** AbbVie Deutschland GmbH & Co. KG, Knollstraße 50, 67061 Ludwigshafen, Germany; barbara.schoenfeld@abbvie.com (B.S.); julius.sundermann@abbvie.com (J.S.); benjamin-luca.keller@abbvie.com (B.-L.K.); oliver.heinzerling@abbvie.com (O.H.)

**Keywords:** nanocrystals, vacuum drum drying, spray drying, drying protectants, redispersibility, compression analysis, ABT-199, solidification

## Abstract

The present study compared vacuum drum drying (VDD) and conventional spray drying (SD) for solidifying crystalline ABT-199 nanosuspensions into redispersible oral drug products. The aim was to optimize formulation compositions and process conditions to maintain nanoparticle size after tablet redispersion. The impact of drug load (22%, 33%, 44%) and type of drying protectant (mannitol, mannitol/trehalose mix (1:1), trehalose) on redispersibility and material powder properties were investigated. Moreover, compression analysis was performed assessing the influence of compaction pressure on primary nanocrystal redispersibility and tablet disintegration. Higher drug loads and lower drying protectant levels resulted in particle growth, confirming a drug load dependence on redispersibility behavior. Notably, all drying protectants showed similar protection properties at properly chosen drying process parameters (*T*_g_-dependent), except when VDD was used for mannitol formulations. Differences between the applied drying processes were observed in terms of downstream processing and tabletability: mannitol-containing formulations solidified via VDD showed an improved processability compared to formulations with trehalose. In conclusion, VDD is a promising drying technique that offers advantageous downstream processability compared to SD and represents an attractive novel processing technology for the pharmaceutical industry. As demonstrated in the present study, VDD combines higher yields with a leaner manufacturing process flow. The improved bulk properties provide enhanced tabletability and enable direct compression.

## 1. Introduction

In recent years, there has been an increased focus on addressing the issue of poorly water-soluble API candidates in pharmaceutical development [1,2]. One strategy to overcome this solubility challenge is the nanocrystal approach, which involves reducing the size of the crystalline API to improve dissolution rate and saturation solubility, in accordance with the Noyes–Whitney and Ostwald–Freundlich principles [1,3,4,5].

There are several FDA-approved products that have utilized the nanocrystal approach, such as Rapamune^®^ (Pfizer, 2000), which was one of the earliest products. Other examples include Invega Sustenna^®^ (Janssen, 2009) and Ryanodex^®^ (Eagle Pharmaceutical, 2014) [6,7]. Furthermore, nanocrystal suspensions are commonly employed in the preclinical stages for toxicological studies, highlighting their importance in early formulation development [8].

Nanocrystal formulations typically consist of an aqueous nanosuspension with particle sizes ranging from 100 to 1000 nm. However, these nanoparticle suspensions require stabilizing excipients due to their high surface energy. There are two types of stabilizers commonly used in the literature: ionic stabilizers for thermodynamic and electrostatic stabilization such as sodium dodecyl sulfate or sodium deoxycholate, and steric stabilizers for kinetic stabilization [9,10]. Steric stabilizers can be classified as non-ionic (e.g., polysorbate 80) and polymeric stabilizers (e.g., copovidone). The most effective stabilization is achieved by combining both types, known as electro-steric stabilization, which exhibits a synergistic effect [11,12].

Basically, there are two main approaches for size reduction techniques: bottom-up and top-down [13]. Top-down approaches involve breaking down large particles into smaller ones using methods like media milling or high-pressure homogenization. Bottom-up approaches involve particle growth or formation through processes like precipitation [14]. However, controlling particle growth in bottom-up approaches is difficult, making them less suitable for larger scales and commercial-scale production in the pharmaceutical industry [14].

Aqueous suspensions of nanocrystals can pose certain risks of instabilities, including physical instabilities like Ostwald ripening, sedimentation, and agglomeration [15]. Moreover, chemical instabilities such as hydrolysis or microbial growth can limit the product shelf life [16]. Additionally, the oral administration of the liquid nanosuspension can lead to dosing errors and reduced patient compliance and requires suitable dosing devices. To address these shortcomings, the liquid nanocrystal suspension can be converted into a solid dosage form, such as tablets or capsules filled with powder. This can be accomplished through drying/solidification techniques like spray drying, spray coating (also referred to as spray granulation), and freeze drying [17,18]. Each drying technique has its own advantages and limitations.

Independent of the drying technique used, drying protectants (also known as bulking agents, dispersants/protectants or embedding matrix material) are commonly added to the crystalline nanosuspension stabilized with ionic/steric stabilizers prior to the thermally stressful drying step to avoid particle growth/agglomeration. Drying protectants are known from lyophilization and are mostly sugars (sucrose, trehalose, or lactose) or sugar alcohols (mannitol) acting as spacers to avoid crystal-to-crystal contact [19]. To ensure the positive impact of nanosizing on dissolution, it is crucial to consider the nanoparticulate redispersibility and the preservation of crystallinity in both the solidified intermediate and final solid dosage form.

In summary, stabilization is necessary in two steps:

(a) To produce a stable nanosuspension using techniques like wet ball milling, where ionic and/or steric stabilizers are employed.
(b) To solidify the nanosuspension through a drying process using drying protectants/bulking agents (such as sugars or sugar alcohols), which act as spacers to prevent crystal-to-crystal contact.

The redispersibility is defined as the amount of the nano proportion after the redispersion of the dried intermediate/final drug product, thus referring to the particle size distribution.

Several methods are available for characterizing nanocrystals in terms of size, but each of them has certain disadvantages regarding accuracy of particle size characterization, when applied as single size characterizing method. Therefore, a combination of at least two methods is recommended [20,21]: (1) indirect particle size characterization applying laser-based methods such as dynamic light scattering (DLS) or laser diffraction (LD) and (2) direct particle size characterization applying microscopic methods such as electron microscopy or light microscopy. Combining the two techniques allows characterization of a very large particle number (laser-based methods) including some visual information on particle shape and size of microscopically visual particles.

LD is the preferred method for characterizing nanocrystals regarding particle size distribution. The LD technique has a broader detection range between approximately 10 nm and 3500 µm, but it has inferior resolution compared with DLS in the lower nanometer range. DLS, on the other hand, is not able to detect particles > 3 µm due to the absence of Brownian motion and the related sedimentation. Additionally, DLS has an inherent difficulty in properly analyzing polydisperse samples. Consequently, inhomogeneous “nanoparticle samples” (consisting of various particle fractions having different particle sizes > 1 µm) cannot be detected accurately using DLS. This limitation of the technique should be considered as it represents an issue when DLS is applied as the only method used to investigate nanoparticle suspensions.

In contrast to laser-based methods, microscopic methods can only provide a very small sample of the total particle population. The resolution of light microscopes is limited to around 200 nm, making light microscopy useful only for particles above 1 µm. Images of particles can be misleading when drawing conclusions about the total particle population. However, in combination with laser-based methods, the images can be helpful to verify and complement the laser-based measurements.

Several published studies reported on the solidification of crystalline nanosuspensions [14,18]. Czyz, Wewers [22] investigated the impact of spray drying temperature, the type of drying, and the API particle size on the redispersibility of naproxen and itraconazole. Wewers, Finke [23] described the formulation parameters on the redispersiblity of naproxen nanoparticles from granules produced in a fluidized bed process. Another study focused on freeze drying, assessing formulation and process factors on the redispersibility of cilostazol nanocrystals [24]. A comparative study on silybin nanosuspension dried via freeze and spray drying was described by Ma, Gao [25]. Fluid bed granulation was also investigated to convert indomethacin nanosuspensions into solid dosage forms [26].

Recently, vacuum drum drying was introduced as a novel drying technique in the manufacturing of ASD-based drug products [27] as well as for the solidification of nanocrystal suspensions [28]. However, an experimental study comparing vacuum drum drying with spray drying as a commonly used drying technique for crystalline nanosuspensions and exploring benefits and distinctions of each technique is missing. In the literature, several studies investigated the manufacturability and dissolution of nanocrystalline-based tablets [26,29]. However, the specific impact of the compaction pressure on the redispersibility of a solidified nanocrystalline intermediate addressing even a mechanistic understanding by considering tablets with tensile strengths higher than usually targeted was not reported.

Thus, the present study compares spray drying (small-scale) with vacuum drum drying (pilot-scale) in terms of manufacturability (yield, visual appearance as, e.g., product spreading on drums (VDD) or string formation (SD)), redispersibility and downstream-processability of resulting intermediates including compression analysis. For this study, solid nanocrystal powders were manufactured at different drug loads (22%, 33%, 44% *w*/*w* (solid)) with different commonly used drying protectants (mannitol, mannitol/trehalose mix (1:1) or trehalose). Czyz, Wewers [22] and Schönfeld, Westedt [28] have previously reported that the glass transition temperature (*T*_g_) of formulations can affect redispersibility, depending on the drying process parameters employed. In order to assess a wide range of *T*_g_ values for the resulting solids, trehalose was selected as a component with a high *T*_g_ (115 °C [30]), mannitol with a low *T*_g_ (87 °C [30]), and a 1:1 mixture with a *T*_g_ in-between. Subsequently, the solid intermediates were characterized for drying effectiveness and the extent to which the nanoparticulate nature of the drug product was preserved after drying. In addition, the impact of tableting on the redispersibility of these solid intermediates was assessed at increasing compaction pressures.

Vacuum drum drying is a continuous drying process where the solution/suspension is being dispensed between the gap of two counter-rotating drums. This leads to the formation of a thin film on both drums, which is subsequently dried through the application of vacuum and drum heating. After approximately three-fourths of a drum turn, the dried film is scraped off by knives. The drying effectiveness relies on various factors including the temperature of the drums, their rotation speed (which determines the duration of residence time), the vacuum conditions, and the formulation properties such as the solid load of the liquid. During spray drying, the feed solution/suspension is sprayed into a hot gas stream to evaporate the solvent. In the pharmaceutical industry, spray drying is a commonly used standard drying process, whereas VDD is rarely employed. However, VDD can offer advantages over SD including cost-effectiveness, higher yields and suitability for early phase development. Additionally, VDD normally eliminates the need for a secondary drying step and does not have viscosity limitations, making it suitable for processing even pastes. This allows for high solid loads in the solution/suspension, reducing solvent and solvent recovery costs.

ABT-199, also known as Venetoclax, was chosen as example compound for this study with the following physicochemical properties (form I): poor water solubility (<4 ng/mL at pH 7.4 in aqueous buffer) [31], melting point of 145 °C and glass transition temperature of 121 °C. Crystalline ABT-199 nanosuspensions were stabilized by sodium deoxycholate and copovidone and were prepared by wet ball milling. ABT-199 is a potent and selective BCL-2 inhibitor approved for CLL (chronic lymphocytic leukemia), SLL (small lymphocytic lymphoma) and acute myeloid leukemia and marketed by AbbVie Inc., North Chicago, IL, USA [32,33,34]. Sodium deoxycholate functions as an ionic stabilizer, while copovidone acts as a polymeric steric stabilizer. When combined, they work synergistically to provide electro-steric stabilization of the nanosuspension during the wet ball milling process. The stabilizer selection was based on previously performed miniaturized formulation screening.

## 2. Materials and Methods

### 2.1. Materials

ABT-199 was obtained from AbbVie Operations Singapore Pte Ltd. (Singapore). Copovidone (polyvinylpyyrolidone-vinyl acetate copolymer, Kollidon^®^ VA64) was purchased from BASF SE (Ludwigshafen, Germany); sodium deoxycholate (origin: animal (bovine/ovine)), mannitol (Parteck M 200 Emprove^®^ Essential) and trehalose dihydrate (Emprove^®^ Expert) were purchased from Merck KGaA (Darmstadt, Germany). Zirconium oxide beads were purchased from Netzsch (Selb, Germany).

### 2.2. Methods

#### 2.2.1. Manufacture of ABT-199 Nanosuspension

The ABT-199 nanosuspension was prepared by wet ball milling in 3 sub-batches each with a batch size of 1.8 kg using the bead agitator mill DeltaVita^®^ 15-300 (Netzsch Feinmahltechnik, Selb, Germany) equipped with a 300 mL grinding chamber and a 2000 mL batch tank. The batch tank as well as the grinding chamber were water-cooled. Before nano milling, the ABT-199 (15% *w*/*w*) suspension was predispersed in a stabilizer-containing aqueous solution (sodium deoxycholate and copovidone) using a magnetic stirrer (IKA GmbH & Co. KG, Staufen, Germany). The composition of the nanosuspension was selected based on results of a previously performed formulation screening (38 different formulation prototype compositions, and is provided in Table 1.

Zirconium oxide beads (size: 0.5 mm) were used as grinding media (bead-to-API-ratio: 3:1). Nano milling was performed under the following conditions: agitator speed 2900 rpm (equal to a tip speed of 10 m/s), pump speed 200 rpm, total milling time 120 min.

After milling, the zirconium beads were separated via a 200 µm mesh size sieve. The yield of each sub-batch was in the range of 86–90%. The three sub-baches were merged using a magnetic stirrer (IKA GmbH & Co. KG, Staufen, Germany).

Prior to subsequent vacuum drum drying or spray drying, the different drying protectants (mannitol, mannitol/trehalose mix (1:1) or trehalose) as well as copovidone (4% *w*/*w*) were dissolved in the ABT-199 nanosuspension while stirring. The amount of drying protectant was adapted for each formulation to meet the final solid composition of 22%, 33% and 44% ABT-199, respectively. The final composition of the dried intermediates is given in Table 2. Besides sugars as drying protectant, copovidone (4% *w*/*w*) was also added to increase the viscosity of the liquid formulation and, thus, to increase the adhesion of the solution to the drums during the vacuum drum drying process. Copovidone was selected since it was already part of the composition as a stabilizer for the nanosuspension preparation. To compare identical formulation compositions processed via VDD or SD, copovidone was also added to the nanosuspension processed via spray drying.

#### 2.2.2. Nanosuspension Characterization—Particle Size Distribution

##### Dynamic Light Scattering

A Zetasizer Ultra (Malvern Instruments GmbH, Herrenberg, Germany) was utilized to measure the z-average and polydispersity index (PDI) of the nanosuspensions via the dynamic light scattering (DLS) method before the drying protectants were added. The hydrodynamic diameter is represented by the z-average, while the PDI characterizes the width of the particle size distribution. The samples were diluted in demineralized water (at a ratio of 1:20), and dilutions were measured in polystyrene single-use cuvettes (DTS0012). The measurements were conducted in backscatter mode (173°) in triplicate at 25 °C following an equilibration period of 120 s. The results were analyzed using ZS Xplorer software (version 2.2.0.147).

##### Laser Diffraction

The particle size distribution of the nanosuspensions was determined using the laser diffraction particle size analyzer Mastersizer 3000 equipped with the automated dispersion module “HydroMV” (Malvern Instruments GmbH, Herrenberg, Germany). A nanosuspension was slowly pipetted to deionized water that was stirred inside the dispersion module tank at 2000 rpm until a laser obscuration of approximately 2 to 3% was achieved. Subsequently, the suspensions were equilibrated in the dispersion module tank for 1 min. The measurement time was set to 10 s both with red light (632.8 nm) and blue light (470 nm). The data obtained were analyzed according to the MIE theory using Mastersizer 3000 Software (version 3.81). The measurements were performed in triplicate, and the results were averaged.

#### 2.2.3. Solidification/Drying of Nanosuspensions

The drying process parameters for spray drying and vacuum drum drying were chosen based on prior knowledge [28] and previously performed process development experiments.

##### Vacuum Drum Drying

A vacuum drum dryer (Buflovak, Buffalo, NY, USA) equipped with a 1 L vessel was used for the drying of the nanosuspensions. The following process parameters were kept constant during drying for all compositions tested: casing temperature 85 °C, gas atmosphere pressure of 100 mbar, drum rotation speed 0.3 rpm, drum gap 0.2 mm. The drum temperature was varied considering the type of drying protectant and the resulting estimated *T_g_* of the solid formulation: the drum temperature was set to 55 °C for mannitol formulations and 75 °C for mannitol/trehalose (1:1) and trehalose formulations. Secondary drying was not required for all VDD intermediates.

##### Spray Drying

Spray drying was performed on a Büchi B-290 laboratory spray dryer (Büchi, Labortechnik GmbH, Essen, Germany) equipped with an inert loop (B-295) and dehumidifier (B-296). A twin-fluid nozzle with 2 mm cap was used (no automatic nozzle cleaning). The rather large nozzle size was chosen to produce larger particles that lead to a potentially better powder flowability. The following parameters were applied to all formulations: feed rate 10 to 12 g/min, nitrogen gas flow 55 mm (approx. 670 L per hour), aspirator 100% (approx. 35 m^3^ per hour), inlet temperature 75 to 85 °C targeting an outlet temperature of 40 °C, inert loop temperature 10 °C.

A secondary drying step was applied to the SD materials using a vacuum tray dryer set to a temperature of 40 °C and to a vacuum of 100 mbar for 20 h to ensure similar moisture levels for SD and VDD material (vacuum oven from Binder GmbH, Tuttlingen, Germany).

#### 2.2.4. Comminution/Deagglomeration

The VDD intermediates were manually comminuted and the SD intermediates manually deagglomerated using an 800 µm mesh size sieve.

#### 2.2.5. Redispersibility of Intermediates and Tablets Using Laser Diffraction

To assess redispersibility, 300 to 450 mg of the SD and VDD powder intermediates were dispersed in deionized water in a 20 mL scintillation vial targeting the initial ABT-199 concentration of the nanosuspension (15% *w*/*w*) using a vortexer (IKA Vortexer VG3, Staufen, Germany) for 60 s.

Tablets were dispersed in 5 mL deionized water in a 20 mL scintillation vial and agitated in a vortexer (IKA Vortexer VG3, Staufen, Germany) for 30 min to enable complete tablet disintegration and dissolution of water-soluble excipients to avoid interference in the particle size measurements. The resulting ABT-199 concentrations after redispersion were as follows: 6.4% *w*/*w* for 22% DL, 9.6% *w*/*w* for 33% DL, 12.8% *w*/*w* for 44% DL. The dispersions were analyzed for particle size using laser diffraction as described in the Laser Diffraction section.

#### 2.2.6. Intermediate Characterization

##### Particle Size Distribution (PSD) of Dry Powder Using Laser Diffraction

The particle size distribution of the VDD and SD intermediates in powder form was analyzed using a laser diffraction particle size analyzer (Mastersizer 3000, Malvern Instruments GmbH, Herrenberg, Germany) equipped with the dry powder disperser module Aero S. Samples of approximately 2 to 5 g were dispersed at 2 bar (*n* = 3). The data obtained were analyzed with Mastersizer 3000 Software (version 3.81), with the application of Mie theory approximation (refractive index 1.644).

##### Powder Bulk/Tapped Density and Particle Density

The powder bulk/tapped density measurements were conducted in accordance with Ph. Eur. 2.9.34 method 1 (measurement in a graduated cylinder). Triplicate measurements were performed.

Particle density was determined using a helium pycnometer (AccuPyc 1340, Micromeritics GmbH, Aachen, Germany) equipped with a 10 cm^3^ sample chamber. The equipment was operated at a cycle fill pressure of 134.45 kPa and an equilibration rate of 0.0345 kPa/min. For each analysis, 5 cycles were performed. All samples were measured as triplicates.

##### Flowability (Ringshear Tester)

The flow properties of the powder intermediates were determined using a ring shear tester (RST-XS, Dietmar Schulze, Schüttgutmesstechnik, Wolfenbüttel, Germany) equipped with a 31.37 mL cell for the VDD material and with a 9.65 mL cell for the SD material. Different cells were used according to the vendor instructions for the respective particle sizes. Triplicate measurements were conducted under the following conditions: pre-shear normal stresses of 0.250, 0.525, 0.800, and 1 kPa at ambient temperature. The data were analyzed using regression analysis.

##### Loss on Drying

The moisture content of the VDD and SD intermediates was determined using a halogen moisture analyzer (HB43-SSD, Mettler-Toledo GmbH, Giessen, Germany) by applying the loss on drying (LOD) method. Samples of approximately 6 g were heated to 105 °C and maintained until a constant mass was achieved within ±1 mg for 100 s. Triplicates were measured for VDD intermediates, and single measurements were performed for SD intermediates due to lack of material (poor yields).

#### 2.2.7. Compression Analysis and Tableting

The HB50 compaction simulator (Huxley Bertram Engineering Limited, Cambridge, UK) was used for compression analysis according to USP <1062> equipped with an 8 mm, round biplane tooling (Euro B) simulating a Korsch XL100 at 30 rpm turret speed. The rather low but reasonable turret speed of 30 rpm was selected to ensure adequate elimination of air from the powder during compression since no outer phase excipients (lubricant/glidant) were added to the intermediates. The target tablet mass was 150 mg. Compaction pressures were varied in the range of 50 to 250 MPa.

Tablets for disintegration and redispersibility testing were manufactured at compaction pressures of 50, 75, 100, 125 and 150 MPa. Tablets were characterized by determining the tablet weight (Sartorius BP 61 S-0CE, Sartorius AG, Goettingen, Germany), the tablet thickness via caliper (Hommel Hercules Werkzeughandel GmbH & Co. KG, Viernheim, Germany), and the tablet diameter and tablet breaking force (Lab.Line H4, Kraemer Elektronik, Darmstadt, Germany). The tensile strength of tablets was calculated as described in USP <1217>.

#### 2.2.8. Disintegration

The disintegration test was conducted following the guidelines stated in Ph. Eur. 2.9.1 (test setup A) using a disintegration tester (ZT 722, Erweka GmbH, Heusenstamm, Germany).

## 3. Results

### 3.1. Particle Size Analysis of the ABT-199 Nanosuspensions

**Figure 1:**
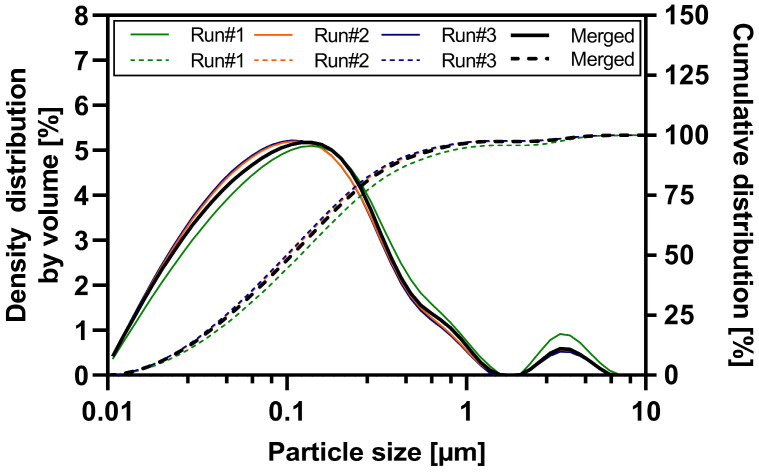
Particle size distribution of nano-suspension sub-batches and merged nanosuspension—density distribution by volume (solid lines) and cumulative distribution (dashed lines).

Wet ball milling of the three ABT-199 sub-batches was performed with high reproducibility and robustness (see Table S2 and Table 3). Each sub-batch contained 1.8 kg of suspension. The particle size distribution data of each sub-batch and the merged nanosuspension determined by laser diffraction is shown in Figure 1. All three sub-batches resulted in nanosuspensions with a d50 value ranging between 99 and 119 nm and a nano proportion (particles < 1 µm in %) of 94.8 to 97.2% (see Table S1). Sub-batch #1 exhibited a slight increase in nanoparticle size compared to sub-batches #2 and #3. This can be attributed to a partial technical failure in water cooling leading to an elevated process temperature during the wet ball milling of sub-batch #1. Product temperatures during milling of the three sub-batches were as follows: 63.9 °C (#1), 53.8 °C (#2), 52.1 °C (#3). A detailed overview of the particle size results of merged ABT-199 nanosuspensions via dynamic light scattering and laser diffraction is given in the supporting information Table S2. The d50 value of the merged nanosuspensions was 109 ± 3.4 nm, and approximately 96.6% of the particles were in the submicron range. The mean z-average value describing the hydrodynamic diameter was 238.6 nm with a polydispersity index of 0.16 indicating a narrow particle size distribution (see Table 3 and Figure S1). Laser diffraction measurements confirmed a narrow particle size distribution in the submicron range and the absence of a substantial population of larger particles > 1 µm (e.g., agglomerates) in the nanosuspension.

### 3.2. Effect of Drying Technique on Solid Yield and on Particle Size upon Redispersion in Water

The impact of the drying techniques spray drying (SD) and vacuum drum drying (VDD) on yield and nano-dispersibility of the intermediates was investigated.

Yield values were consistently higher for the VDD process, ranging from 78 to 94%, compared to SD (15 to 89%), as shown in Table S3. The highest yield values for VDD were observed for mannitol-containing formulations (either pure mannitol or mannitol/trehalose mix). The mannitol-containing formulations showed a flake-like appearance on the drum surface, whereas trehalose formulations were scrapped of the drums as fluffy powder partially sucked in into the exhaust gas stream. The yield of mannitol/trehalose formulations dried via SD showed lowest yield values, ranging from 15 to 40%.

**Figure 2:**
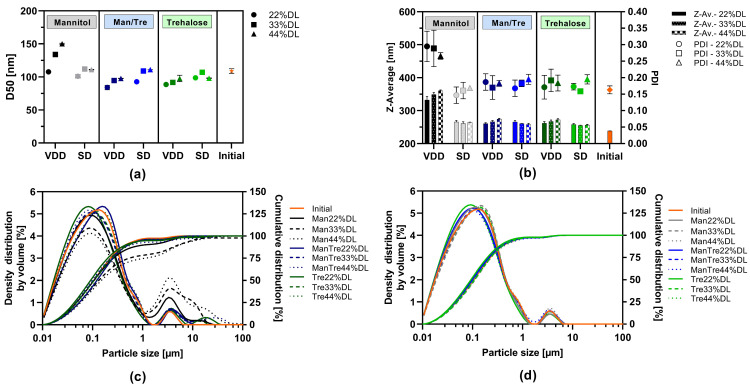
Redispersibility of VDD and SD intermediates (powder), mean particle size results: (**a**) d50 values measured by laser diffraction and (**b**) z-average values and polydispersity index (PDI) measured by dynamic light scattering; density distribution by volume and cumulative size distribution by laser diffraction of intermediates redispersed compared to initial nanosuspension before drying: (**c**) VDD and (**d**) SD.

The impact of the drying protectant (mannitol, mannitol/trehalose mix or trehalose) and ABT-199 drug load within the solid intermediate was assessed with respect to particle size (via LD and DLS) and PDI after the redispersion of the SD or VDD powder in water (redispersibility). The results are displayed in Figure 2. The d50 values ranged from 84 to 111 nm and were in a similar range to the initial nanosuspension after milling for all formulations except for the VDD_Man_33% and VDD_Man44% formulations (see Figure 2a). Formulations VDD_Man_33% and VDD_Man_44% showed higher d50 values indicating agglomeration during the VDD drying process, and thus, larger particles were detected within the redispersed suspension of the VDD intermediate (see Figure 2c). Figure 2c also reveals an increase in the proportion of larger particles for the VDD-Man_22% formulation, although this is not indicated by the d50 value. In contrast, all SD formulation distribution curves (see Figure 2d) were comparable with the one of the initial nanosuspension.

Moreover, a slight trend towards higher d50 values was observed under most conditions, correlating with increasing drug load and, thus, correlating with decreasing drying protectant proportion.

The z-average values ranged between 255 and 274 nm for the mannitol/trehalose mix or trehalose-containing formulations dried by SD or VDD (see Figure 2b). However, an impact of drying technique on z-average values was observed for the mannitol-containing formulations processed by VDD resulting in an increase in particle size and a broad polydisperse particle size distribution expressed as a PDI of 0.3. In contrast, SD intermediates consistently showed similar z-average values across all formulations irrespective of drug load and used drying protectant type.

### 3.3. Characterization of VDD and SD Intermediates

**Figure 3:**
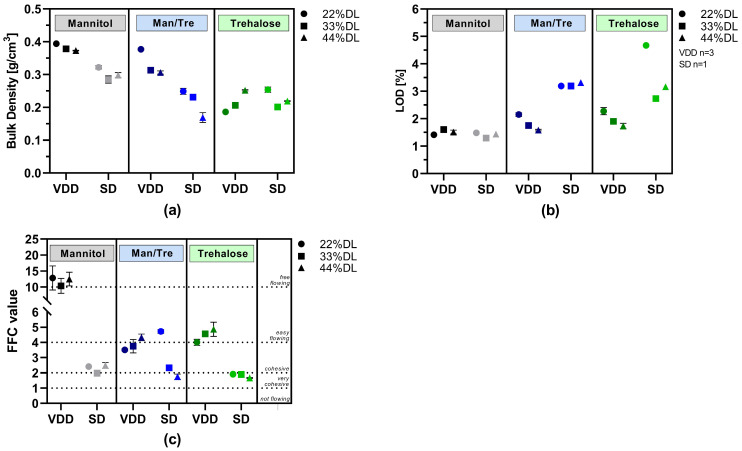
Powder characterization results: VDD and SD intermediates consisting of different ABT-199 drug loads: (**a**) bulk density; (**b**) loss on drying; (**c**) flowability as flow function coefficient (FFC) (>10: free-flowing; 4–10: easy-flowing; 2–4: cohesive; 1–2: very cohesive; <1: not flowing).

The following bulk properties of the dried intermediates were characterized: loss on drying (LOD), density (particle, bulk/tapped), flowability (ring-shear testing) and solid particle size distribution (PSD, via laser diffraction). The results are summarized in detail in Table S3 and visualized in Figure 3.

Bulk density results are visualized in Figure 3a. Mannitol and mannitol/trehalose-containing formulations dried via VDD exhibited the highest bulk densities, around 0.3 to 0.4 g/cm^3^. In contrast, the bulk density of the mannitol and mannitol/trehalose SD intermediates was lower, ranging from 0.17 to 0.32 g/cm^3^, indicating up to a 50% difference in bulk density. Similar bulk densities were observed for trehalose-containing formulations independently of the applied drying technique (0.17 to 0.25 g/cm^3^).

Although no secondary drying was conducted, VDD intermediates showed low LOD values between 1.4 and 2.3% (Figure 3b). The LOD values for the SD intermediates were slightly higher, ranging from 1.4 to 4.7%, despite the additional secondary drying after spray drying. In particular, the trehalose-containing SD formulations showed substantially higher moisture content (2.7–4.7%).

The flow properties of the SD and VDD intermediates were characterized using a ring shear tester. The resulting flow function coefficients (FFCs) are shown in Figure 3c. In all cases, the VDD mannitol formulations exhibited free-flowing properties, whereas the respective SD formulations showed cohesive flow. Easy-flowing properties were observed for trehalose VDD intermediates, but only very cohesive flow behavior was observed for SD intermediates.

**Figure 4:**
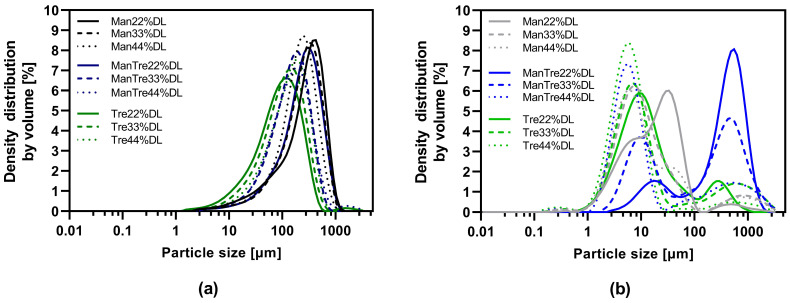
Particle size distribution of dry powders via laser diffraction: (**a**) VDD intermediates comminuted with an 800 µm mesh sieve; (**b**) SD intermediates deagglomerated with an 800 µm mesh sieve.

The particle size distribution is listed in Table S3 and visualized in Figure 4. The results indicated a monomodal distribution for all VDD intermediates and a bimodal distribution for the SD intermediates. The proportion of small particles (<10 µm) was larger for the SD intermediates in combination with an elevated residual moisture content due to the quite low outlet temperature, this resulted in a high agglomeration tendency explaining the bimodal distribution. The d50 values for the pure mannitol containing VDD intermediates were in the range of 210 to 294 µm, for mannitol/trehalose formulations in the range of 149 to 250 µm, and for pure trehalose in the range of 93 to 141 µm (Figure 4a, Table S3). In contrast, the d50 values for SD intermediates were much smaller, in a range between 6 and 19 µm for all formulations except for SD_Man/Tre_22% (388 µm) and SD_Man/Tre_33% (171 µm) (Figure 4b, Table S3).

### 3.4. Compression Analysis of VDD and SD Intermediate Powders—Tabletability

**Figure 5:**
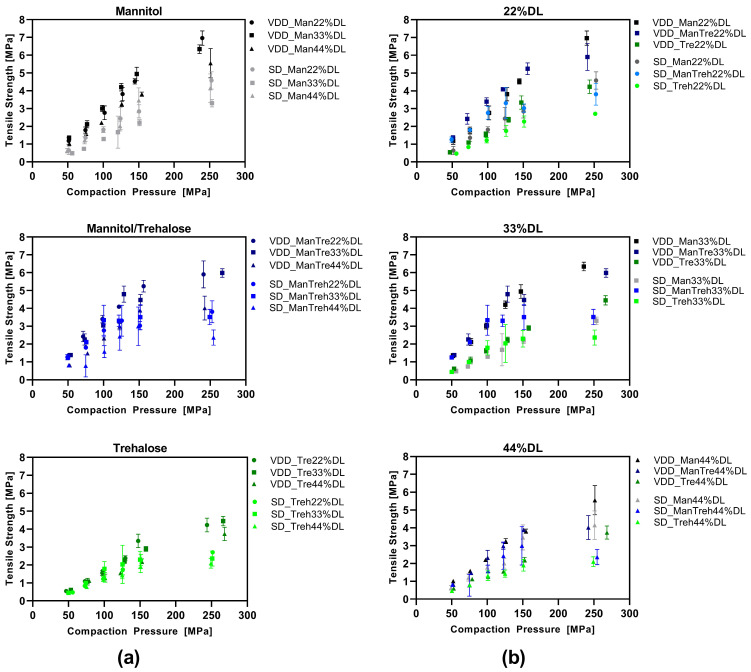
Tabletability—simulating Korsch XL100 at 30 rpm turret speed: (**a**) grouped by drying protectant used comparing VDD and SD formulations at different ABT-199 drug loads; (**b**) grouped by ABT-199 drug loads comparing VDD and SD formulations containing different drying protectants.

The tabletability of the dried intermediates was analyzed by plotting the tensile strength as a function of the compaction pressure (Figure 5). VDD and SD intermediates were compared with respect to the type of drying protectant used (Figure 5a) and the drug load (Figure 5b). Overall, the VDD intermediates exhibited better tabletability than the SD intermediates. The tabletability of the mannitol-containing formulation was enhanced at applied compaction pressures, as evidenced by the higher maximum tensile strength (VDD: 6–7 MPa; SD: 4–4.5 MPa) compared to the trehalose formulations (VDD: 4 MPa; SD: 2.5 MPa). An increased drug load led to lower tensile strengths independent of the applied drying process.

Besides the tabletability, the VDD and SD intermediates also exhibited differences in terms of manufacturability. Whereas the VDD intermediate powders were manufactured using the automatic feeder of the compaction simulator, the SD intermediates needed to be filled manually into the die due to their poor flow and unfavorable electrostatic behavior.

More detailed compression analysis is visualized in the Supplementary Materials (compactability plots in Figure S2, compressibility plots in Figure S3, elastic work plots in Figure S4).

### 3.5. Impact of Tableting on Nanoparticle Size upon Redispersion in Water

**Figure 6:**
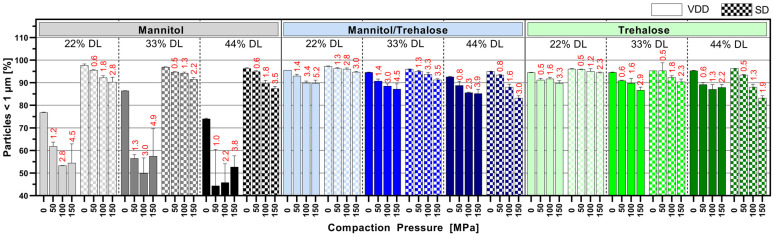
Mean particle size results of tablets manufactured at different compaction pressure values (50, 100 and 150 MPa, 0 MPa = powder)—number of particles below 1 µm in % (nano proportion) determined via laser diffraction (red numbers: mean tensile strength at respective compaction pressure).

Figure 6 displays the nano proportion (particles < 1 µm in %) after the redispersion of tablets manufactured at 50, 100 and 150 MPa compared to redispersed intermediate powder (0 MPa). Interestingly, for the mannitol/trehalose or trehalose intermediates, there was only a modest influence of the compression process on the redispersibility of the nanoparticles. The mannitol/trehalose or trehalose formulations manufactured by VDD and SD exhibited a redispersed nano population > 85% even at a high compaction pressure > 100 MPa. But the data revealed a clear trend towards decreasing nano proportions with increasing compaction pressure independent of the drying process. The correlation between compaction pressure on a solid nanoparticle dispersion and the increase in particle size after redispersion has to our knowledge not yet been described. However, it might be correlated to elastic and plastic deformation associated cohesion effects between individual nanoparticles. This impact was less pronounced for 22% drug-loaded formulations and, thus, for formulations containing the highest amount of drying protectant. VDD mannitol formulations showed a strong loss of the nano-sized ABT 199 population (particles in % <1 µm) after compression, with the maximum decrease to 50% (for 44% DL). However, the redispersed powder values for the VDD mannitol formulations (0 MPa, without compression) already indicated a certain quality loss evident as a reduction in the nano population (~75–85% <1 µm). In contrast, SD mannitol intermediates showed a lower loss of the nano proportion, maintaining nano proportions similar to those of the mannitol/trehalose or trehalose SD intermediates.

The respective mean tensile strengths of the tablets are noted above the bars in red (see Figure 6), showing a huge difference in mechanical strength of the assessed tablets.

### 3.6. Disintegration

**Figure 7:**
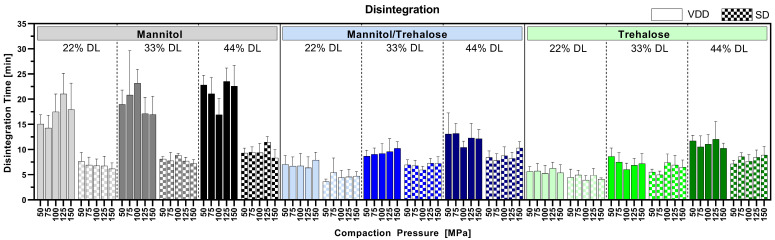
Disintegration time of tablets compressed at 50, 75, 100, 125 and 150 MPa compaction pressure (varying tensile strength depending on compactability of intermediates).

Figure 7 shows the impact of compaction pressure ranging from 50 to 150 MPa on tablet disintegration for all tested formulations. The VDD-derived pure mannitol-containing tablets showed a disintegration time below 25 min, whereas all other compressed VDD intermediates disintegrated below 12.5 min independent of the applied drying technique. VDD intermediates showed either similar or slower disintegration compared to SD intermediates. An impact of compaction pressure on disintegration time was not observed within the tested range. However, a slight trend towards higher disintegration time with increasing drug load was observed, which might be related to the decreasing proportion of water-soluble components (drying protectant), which normally supports tablet disintegration.

## 4. Discussion

This study comparing a commonly used spray drying technique versus a vacuum drum drying technique for the solidification of nanocrystals revealed three main findings. First, the drying technique can impact the nanoparticulate nature and related redispersibility behavior and affects the bulk properties of the intermediates when the same formulation compositions are compared to each other. Second, varying the drug load and the related changes in excipient levels to stabilize the nanoparticles during drying affects the redispersibility of the solid intermediates by influencing the glass transition temperature of the formulation. But the results suggested that this impact can be mitigated by applying the appropriate processing temperature and not exceeding the glass transition temperature of the formulation during drying. Third, the drying technique impacts the bulk properties and related compression behavior and therewith the downstream processability of the nanocrystal-based solid intermediate to a tablet.

Overall, an ABT-199 nanocrystalline suspension was successfully manufactured with good reproducibility through a wet ball milling technique utilizing zirconium oxide beads in a pilot-scale bead agitator mill. The d50 and d90 values were in a comparable range for all three sub-baches, indicating the desired quality for a liquid nanosuspension (d90 < 1 µm targeted) before drying to a solid intermediate via spray drying or VDD.

Redispersibility behavior and maintaining the nanoparticulate nature in the solid intermediates seem to be a complex interplay between applied drying technique as well as drug load in the formulation and drying protectant type used. In an assessment of the impact of drug load (22%, 33%, 44%) and the respective drying protectant types and proportions within the formulation on redispersibility, the data implied that redispersibility was mainly influenced by the type of drying protectant and the drying technique itself. The impact of the drying technique was already described by Czyz, Wewers [22] for naproxen and itraconazole, where the spray drying process boundary (outlet temperature) for redispersibility to a nanocrystalline suspension correlates with the glass transition temperature of the pure drying protectant, as shown for trehalose, sucrose, and lactose. Similar observations were made by Schönfeld, Westedt [28] for vacuum drum drying in an investigation of mannitol, trehalose, and lactose as drying protectants for a ritonavir nanosuspension. Therefore, a lower process temperature in VDD was initially applied for the mannitol-only (55 °C) compared to the mannitol/trehalose or trehalose-only formulation (75 °C) because the glass transition temperature of mannitol is lower than that of trehalose [30]. However, the drum temperature of 55 °C for the mannitol formulation was obviously above the critical temperature in the present study because the particle size data of the redispersed VDD intermediate suggest the formation of agglomerates/particle growth. Consequently, the drying of the mannitol formulation was repeated at an even lower drum temperature of 45 °C. But at this temperature, the nanosuspension showed less adherence to the drum surface, so the nanosuspension could not be dried. At a drum temperature of 50 °C, the nanosuspension was processed, but again, redispersibility properties were not enhanced. In contrast, SD mannitol formulations demonstrated good redispersibility and maintained their initial PSD after nano milling in this study. The preservation of the nanoparticulate nature/size can be explained by the lower outlet temperature (40 °C) during spray drying and shorter residence time of each particle within the spray dryer, resulting in reduced exposure to thermal stress during drying. The mean residence time in a Büchi B-290 spray dryer is about 1.0 to 1.5 s according to the vendor’s technical datasheet [35]. In contrast, the VDD drum speed was set to 0.3 rpm, resulting in a considerably longer mean residence time of approximately 120 to 150 s.

However, a slight trend towards a loss of nano proportion with increasing ABT-199 drug load was observed in the present study for both drying techniques and for all drying protectant types used. Because the amount of drying protectant in the formulation composition decreases with an increase in the API load, nanoparticle aggregation might be facilitated by the reduced spacer effect of the drying protectant as Zuo, Sun [36] described previously. According to their explanation, water-soluble additives like mannitol can create hydrophilic bridges between the nanoparticles to prevent crystal-to-crystal contact and ultimately crystal growth. In addition, Wewers, Finke [23] showed that there is a correlation between the concentration-dependent nanoparticle distance within a solid intermediate and its nano-dispersibility. This same stabilizing concentration-dependent effect can be observed in the lyophilization processes of nanoparticles, where sugar is utilized as a so-called “cryo-protectant” to prevent agglomeration by acting as a spacer [37]. Moreover, Malamatari, Somavarapu [38] described the mannitol-to-drug ratio as a critical parameter affecting redispersibility, and similar observations were made by Sun, Ni [39], explaining the decrease in nano proportion in the presented study.

In the present case, the most favorable ABT-199 drug load for the target final dosage form “tablets” seems to be 22% considering redispersibility. Often, formulations with high drug loads are desired to minimize pill burden for patients, especially if high dosage strengths are required. Despite the feasibility of manufacturing high-drug-loaded nanocrystalline powders, the redispersibility of resulting tablets might not be given to its full extent since the applied compaction pressure may destroy the nanoparticulate structure by resulting in agglomeration. However, the impact of a minor loss of the nano proportion (e.g., down to 80–90%) due to compression on bioavailability has not been extensively studied yet. Many studies assessed the dissolution of nanocrystal-based tablets without confirming their quality in terms of nano-dispersibility after compression. For instance, dissolution profiles were beneficial for an itraconazole nanocrystal formulation dried via spray drying compared to a microcrystalline formulation [30]. Li, Zhou [40] demonstrated that tablets consisting of loratadine nanocrystals showed similar or better dissolution behavior, depending on the dissolution medium used, and favorable bioavailability compared to a marketed formulation and crude drug tablet. But the presence of nanocrystals and the exact nanocrystalline proportion after redispersion of manufactured tablets has not been assessed in both cases.

The data presented here indicated a certain impact of compaction pressure resulting in poorer redispersibility. In particular, for the 44% drug-loaded formulations with trehalose or mannitol/trehalose, it was discovered that a higher compaction pressure results in lower redispersibility due to agglomeration of the nanocrystals. However, when comparing different drug loads and drying protectants in formulations processed by different drying techniques to each other, it needs to be considered that applied compaction pressures resulted in different tensile strength values. Thus, the respective tablet formulations have different mechanical strengths which may impact the redispersibility and the proportion of particles maintaining their nanoparticulate nature. Thus, a direct comparison of applied compaction pressure versus redispersibility behavior needs to consider the difference in the tensile strength of the tested tablets. In all cases, the compression of VDD intermediates led to higher tensile strength values at tested compaction pressures. Since the mechanical strength of a tablet is correlated with the inner porosity of a tablet (also in the present case, see Figure S2), the propensity for crystal-to-crystal contact is higher for tablets with higher mechanical strengths. A tensile strength of approximately 1.5 MPa is the desired target for drug product development. In most SD and VDD tablets, a tensile strength of ~1.5 MPa was already achieved at low compaction pressures of 50–100 MPa. In contrast to compaction pressures > 100 MPa, the application of a compaction pressure of 50–100 MPa showed no or only minor impact on the redispersed nano proportion (particles <1 µm: >80%). Interestingly, as soon as the solid intermediate showed poor redispersibility after drying like the VDD pure mannitol formulations, the impact of compaction pressure on redispersibility was even more pronounced. Consequently, it seems to be crucial to generate a physically stable solid intermediate first where the nanoparticulate structure is fully maintained after drying. The impact of target tensile strengths up to 2.0 MPa has been assessed by Schönfeld, Westedt [28] for solidified ritonavir nanocrystals. In contrast to the present study, there was no impact of the tableting process on redispersibility observed for tensile strengths up to 2.0 MPa. Although the risk of agglomeration may theoretically increase with increasing compaction pressure, the threshold where the tableting process affects the redispersibility of nanocrystals seems to be formulation- and API-specific and therefore needs to be considered in drug product development. Similar API-specific characteristics for redispersibility were observed for granules loaded with nanoparticles by Wewers, Finke [23]. They reported a minimum mean nanoparticle distance to avoid agglomeration specifically for itraconazole (100 nm) and naproxen (80 nm). This minimum mean nanoparticle distance might be dominated by API particle morphology factors, such as shape or size, and physicochemical properties. Future studies should also focus on evaluating the reproducibility of the tableting process, especially on larger-scale equipment in a long-term run to obtain a comprehensive understanding of the tableting process for solidified nanocrystals. However, to avoid tableting and related redispersion issues of the nano proportion, encapsulation is known to be a less stressful unit operation applying no or only minor compaction pressures. Thus, a powder-filled capsule can be considered as an alternative as a final dosage form unit for nanocrystal products.

In a comparison of downstream processability to tablets of intermediates manufactured either via SD or VDD, the VDD intermediates showed generally favorable powder bulk properties such as improved flowability, lower LOD values after drying and higher bulk density. The SD intermediates consisted of small, spherical particles as is common for the spray drying process [41,42]. Such particles exhibit a high agglomeration tendency as a solid indicated by a bimodal particle size distribution (Figure 4b) and unfavorable electrostatic behavior. These factors contribute to poor flowability and low bulk density. In contrast, VDD intermediates were larger and more platelet-like as observed in previous studies [27]. The VDD bulk density for ABT-199 intermediates was in the similar range as previously reported for solidified ritonavir nanocrystal intermediates processed with similar drying protectants [28]. This suggests that the powder properties might be mainly affected by the drying protectant type and drying technique, with the API having less impact. Despite the absence of secondary drying, the LOD values, and consequently the moisture content values for the VDD intermediates, were significantly lower compared to the spray-dried ones. This difference can be attributed to the longer total drying time in the VDD process compared to the SD process (VDD: 120–150 s, SD: 1.0–1.5 s, see above) and the selected SD process conditions with a relatively low outlet temperature.

The VDD intermediates demonstrated enhanced manufacturability in terms of die filling due to their favorable bulk properties in comparison to the SD intermediates. Furthermore, the VDD intermediates exhibited improved tabletability characterized by higher tensile strength values at comparable compaction pressures and the absence of tablet defects, as demonstrated by the compression analysis. This superior tabletability for the VDD intermediate was unexpected since the bulk particle size of the SD intermediates was much smaller than that of the VDD intermediates. A potential explanation could be that the irregular-shaped particles enable mechanical interlocking during compression combined with plastic deformation of the polymeric components. In future studies, measurements of the specific surface area would be of interest to see if there are differences in total surface area and, thereby, total bonding area.

The LOD values for pure mannitol-containing formulations were comparable for SD and VDD but much higher for mannitol/trehalose SD intermediates compared to VDD. Usually, a water content in a certain range improves the tabletability of a bulk material compared to dryer bulk materials [43,44]. As shown by Sun [43] for microcrystalline cellulose, the optimum water content considering tensile strength was between 3.3 and 5.6% moisture. However, tabletability was improved independent of the type of drying protectants for VDD intermediates, indicating that water content is less likely to be the primary factor and the lower water content of the VDD intermediates did not adversely affect tabletability. Furthermore, tablets containing mannitol exhibited the highest tensile strength and mechanical strength. Mannitol is a commonly used tablet binder in the pharmaceutical industry due to its favorable binding properties [45], while trehalose is not typically employed for this purpose. The tabletability generally decreased as the drug load increased, which can be attributed to the fact that the API has inferior compactability properties in comparison to the drying protectant, even though the proportion of copovidone, a good binder, also increased with increasing drug load [46,47]. A potential explanation is that crystalline structures require higher compaction pressures compared to amorphous sugar-based drying protectants, which were dissolved in the nanosuspension before drying.

The tablet disintegration time was mainly influenced by the type of drying protectant resulting in the following disintegration time rank order: trehalose < mannitol/trehalose mix < mannitol. The disintegration time was less affected by the final drug load of the formulation or the applied drying process. Interestingly, VDD mannitol formulations showed slower disintegration compared to mannitol/trehalose mix or trehalose formulations. This can be explained by the interplay of several factors. First, both drying protectants are soluble in water, with mannitol having higher water solubility compared to trehalose (mannitol: 180 mg/mL, trehalose: 50 mg/mL) [48]. However, a long residence time on the drums for mannitol formulations with a low glass transition temperature might lead to a softening and densification of the material itself on the drums. Thus, high-density particles might decelerate the disintegration due to the reduced surface area/porosity. Second, the present study showed larger particle sizes for VDD mannitol formulations compared to SD mannitol intermediates, resulting in a decreased surface area and inner tablet microstructure correlating even more with an increased disintegration time. The impact of particle size (d90) on disintegration time for different mannitol grades has been described by Kosugi, Leong [49], whereas Skelbæk-Pedersen, Al-Sharabi [50] observed a difference in water ingress into tablets for different particle sizes of the powder particles based on the predominant powder deformation behavior during compression. Third, tablet hardness is known to impact disintegration [51]. Therefore, the disintegration times of VDD mannitol tablets are increased due to their highest tensile strengths amongst all others at tested compaction pressures. Fourth, different polymorphs of mannitol might be present in the VDD and SD intermediates in different proportions, potentially influencing the solubility of mannitol in water and therefore influencing the disintegration time. Yang, Liu [52] described the impact of processing technique on the polymorphism of mannitol and its impact on physicochemical properties such as solubility. Last, the slight trend towards slower disintegration time with increasing ABT-199 drug load might be related to the decreasing proportion of the water-soluble drying protectant. As the amount of water-insoluble ABT-199 proportion increased, the wettability of the tablet was reduced, resulting in a slower disintegration process. Drying protectants are known to improve wettability [53], leading to enhanced water intake and disintegration. Nevertheless, all intermediates, except for the VDD mannitol ones, were within the USP/Ph. Eur. specification for uncoated immediate-release tablets. Overall, disintegration is not fully understood so far [54] and is affected by a complex interplay of powder bulk properties, process parameters/manufacturing techniques, tablet properties and tablet microstructure as stated by Sun [55]. It can be assumed that the presence of nanocrystals adds another variable affecting the disintegration of tablets.

## 5. Conclusions

The present study demonstrated the applicability of vacuum drum drying (VDD) for the solidification of ABT-199 nanocrystalline suspension into a solid nano-dispersible powder as an alternative to the commonly used spray drying (SD) process.

Maintaining the nanoparticulate nature upon drying was identified as a key quality attribute of the solid nanocrystal-based intermediates in a comparison of both drying techniques using different drying protectants at varying drug loads. Except for vacuum drum drying of mannitol formulations, the redispersibility of intermediates showed that nanoparticulate nature was maintained in both drying techniques at specific process parameters for formulations containing mannitol, a mannitol/trehalose mix or trehalose as a drying protectant. It was discovered that the redispersibility was slightly worse with a decreasing proportion of drying protectants (and increasing drug load) within the formulation. In contrast to VDD, SD intermediates showed good redispersibility and maintained the nanoparticulate structure independent of the applied drying protectant type or amount in the formulation (and correlating drug load 22–33–44%). Thus, the shorter drying process of SD (SD: approx. 1.0–1.5 s; VDD: approx. 120–150 s) is beneficial for avoiding thermal stress to the nanoparticles. The lower thermal stress may enable the achievement of a re-nanodispersible intermediate with higher drug load levels (>44%) compared to VDD. Furthermore, the SD intermediates do not require an additional comminution step for downstream processing but rather a sieving/deagglomeration step since the primary particle size of SD intermediates is essentially smaller compared to VDD intermediates. A subsequent sieving/deagglomeration step generates less mechanical stress and may be less stressful for the nanoparticles compared to a comminution step as applied to VDD intermediates.

The redispersibility of tablets was impacted by the compression process and can result in a loss of nano proportion depending on the intermediate drug load as well as the drying protectant type. For the redispersibility of SD and VDD tablets, compaction pressure during tableting and an increase in the size of nanoparticles after redispersion were correlated, while the disintegration was not impacted. Although SD and VDD intermediates behaved similarly in relation to redispersibility, it was clearly shown that processing via VDD has benefits for downstream processing. VDD processing resulted in more favorable bulk properties like improved flowability, higher bulk density and finally better tabletability at a given manufacturability due to constant automatic die filling. Overall, VDD intermediates containing the mannitol/trehalose mix as a drying protectant combined good redispersibility with good downstream processability by means of good flowability, high bulk density and good tabletability. However, in terms of tablet disintegration, the SD tablets revealed generally faster disintegration compared to the VDD tablets, which may result in faster dissolution after the tablet release compared to the VDD tablets. Overall, comparing VDD to SD, the data indicated further benefits for VDD like higher yield values despite the small batch size for a pilot-scale machine and no need for a secondary drying step, and the particle size distribution is more definable by subsequent milling step.

In conclusion, high-drug-loaded (44%) ABT-199 nanoparticulate tablet formulations processed via VDD or SD were feasible in terms of drying and compression but also maintained the nanoparticulate nature in the final dosage form unit. However, considering the impact of the tableting process on the redispersibility, targeting lower drug loads, and applying low compaction pressures are highly recommended to reduce the risk of agglomeration during processing.

## Figures and Tables

**Table 1:** Nanosuspension composition.

Material	Function	Amount [% *w*/*w*]
ABT-199	Active pharmaceutical ingredient	15.00
Sodium deoxycholate	Electrostatic stabilizer	0.85
Copovidone	Steric stabilizer	2.55
Demineralized water	Dispersion media	81.60

**Table 2:** Compositions of dried intermediates containing ABT-199 in 22%, 33% or 44% drug load.

	Mannitol			Mannitol/Trehalose			Trehalose		
	22% DL	33% DL	44% DL	22% DL	33% DL	44% DL	22% DL	33% DL	44% DL
ABT-199	22.00	33.00	44.00	22.00	33.00	44.00	22.00	33.00	44.00
Sodium deoxycholate	1.25	1.87	2.49	1.25	1.87	2.49	1.25	1.87	2.49
Copovidone	12.53	16.79	21.06	12.53	16.79	21.06	12.53	16.79	21.06
Mannitol	64.23	48.33	32.45	32.12	24.17	16.23	-	-	-
Trehalose	-	-	-	32.12	24.17	16.23	64.23	48.33	32.45

**Table 3:** Particle size results of merged ABT-199 nanosuspension using dynamic light scattering.

	Z-Average [nm]	PDI
1	239.4	0.15
2	237.4	0.18
3	238.3	0.16
4	242.2	0.13
5	238.4	0.18
6	235.7	0.16
Mean	238.6	0.16
SD	2.1	0.02

## Data Availability

The data presented in this study are available in the research article.

## Supplementary Materials

The following supporting information can be downloaded at https://www.mdpi.com/article/10.3390/pharmaceutics16060782/s1: Figure S1: Intensity-based particle size distribution of initial nanosuspension (merged nanosuspension of runs #1–3); Figure S2: Compactability—simulating KorschXL100 at 30 rpm turret speed: (a) grouped by drying protectant used comparing VDD and SD formulations at different ABT-199 drug loads; (b) grouped by ABT-199 drug loads comparing VDD and SD formulations containing different drying protectants; Figure S3: Compressibility—simulating KorschXL100 at 30 rpm turret speed: (a) grouped by drying protectant used comparing VDD and SD formulations at different ABT-199 drug loads; (b) grouped by ABT-199 drug loads comparing VDD and SD formulations containing different drying protectants; Figure S4: Elastic work—simulating KorschXL100 at 30 rpm turret speed: (a) grouped by drying protectant used comparing VDD and SD formulations at different ABT-199 drug loads; (b) grouped by ABT-199 drug loads comparing VDD and SD formulations containing different drying protectants; Table S1: Particle size results of ABT-199 nanosuspension sub-batches by laser diffraction; Table S2: Particle size results of merged ABT-199 nanosuspension using dynamic light scattering and laser diffraction; Table S3: Results of drying process and intermediate characterization (LOD = loss on drying; FFC = flow function coefficient).

## Abbreviations

API: active pharmaceutical ingredient; CP: compaction pressure; DLS: dynamic light scattering; FDA: Food and Drug Administration; FFC: flow function coefficient; LD: laser diffraction; LOD: loss on drying; Man: mannitol; PDI: polydispersity index; PSD: particle size distribution; SD: spray drying; Tre: trehalose; TS: tensile strength; VDD: vacuum drum drying; ASD: amorphous solid dispersion.

## References

[1] Khan K.U., Minhas M.U., Badshah S.F., Suhail M., Ahmad A., Ijaz S. (2022). Overview of nanoparticulate strategies for solubility enhancement of poorly soluble drugs. Life Sci..

[2] Lipinski C.A., Lombardo F., Dominy B.W., Feeney P.J. (1997). Experimental and computational approaches to estimate solubility and permeability in drug discovery and development settings. Adv. Drug Deliv. Rev..

[3] Merisko-Liversidge E., Liversidge G.G. (2011). Nanosizing for oral and parenteral drug delivery: A perspective on formulating poorly-water soluble compounds using wet media milling technology. Adv. Drug Deliv. Rev..

[4] Otto D., De Villiers M. (2009). Physicochemical Principles of Nanosized Drug Delivery Systems.

[5] Patravale V.B., Date A.A., Kulkarni R.M. (2004). Nanosuspensions: A promising drug delivery strategy. J. Pharm. Pharmacol..

[6] Patra J.K., Das G., Fraceto L.F., Campos E.V.R., Rodriguez-Torres M.d.P., Acosta-Torres L.S., Diaz-Torres L.A., Grillo R., Swamy M.K., Sharma S. (2018). Nano based drug delivery systems: Recent developments and future prospects. J. Nanobiotechnol..

[7] Jermain S.V., Brough C., Williams R.O. (2018). Amorphous solid dispersions and nanocrystal technologies for poorly water-soluble drug delivery—An update. Int. J. Pharm..

[8] Li P., Zhao L. (2007). Developing early formulations: Practice and perspective. Int. J. Pharm..

[9] Liu P., Rong X., Laru J., van Veen B., Kiesvaara J., Hirvonen J., Laaksonen T., Peltonen L. (2011). Nanosuspensions of poorly soluble drugs: Preparation and development by wet milling. Int. J. Pharm..

[10] Choi J.-Y., Yoo J.Y., Kwak H.-S., Uk Nam B., Lee J. (2005). Role of polymeric stabilizers for drug nanocrystal dispersions. Curr. Appl. Phys..

[11] Lestari M.L.A.D., Müller R.H., Möschwitzer J.P. (2015). Systematic Screening of Different Surface Modifiers for the Production of Physically Stable Nanosuspensions. J. Pharm. Sci..

[12] Yu F., Chen Y., Liang X., Xu J., Lee C., Liang Q., Tao P., Deng T. (2017). Dispersion stability of thermal nanofluids. Prog. Nat. Sci. Mater. Int..

[13] Ahmadi Tehrani A., Omranpoor M.M., Vatanara A., Seyedabadi M., Ramezani V. (2019). Formation of nanosuspensions in bottom-up approach: Theories and optimization. DARU J. Pharm. Sci..

[14] Möschwitzer J.P. (2013). Drug nanocrystals in the commercial pharmaceutical development process. Int. J. Pharm..

[15] Li J., Wang Z., Zhang H., Gao J., Zheng A. (2021). Progress in the development of stabilization strategies for nanocrystal preparations. Drug Deliv..

[16] Wu L., Zhang J., Watanabe W. (2011). Physical and chemical stability of drug nanoparticles. Adv. Drug Deliv. Rev..

[17] Jacob S., Nair A.B., Shah J. (2020). Emerging role of nanosuspensions in drug delivery systems. Biomater. Res..

[18] Malamatari M., Somavarapu S., Taylor K.M.G., Buckton G. (2016). Solidification of nanosuspensions for the production of solid oral dosage forms and inhalable dry powders. Expert Opin. Drug Deliv..

[19] Patel D., Zode S.S., Bansal A.K. (2020). Formulation aspects of intravenous nanosuspensions. Int. J. Pharm..

[20] Gaumet M., Vargas A., Gurny R., Delie F. (2008). Nanoparticles for drug delivery: The need for precision in reporting particle size parameters. Eur. J. Pharm. Biopharm..

[21] Chogale M.M., Ghodake V.N., Patravale V.B. (2016). Performance Parameters and Characterizations of Nanocrystals: A Brief Review. Pharmaceutics.

[22] Czyz S., Wewers M., Finke J.H., Kwade A., van Eerdenbrugh B., Juhnke M., Bunjes H. (2020). Spray drying of API nanosuspensions: Importance of drying temperature, type and content of matrix former and particle size for successful formulation and process development. Eur. J. Pharm. Biopharm..

[23] Wewers M., Finke J.H., Czyz S., Eerdenbrugh B., John E., Büch G., Juhnke M., Bunjes H., Kwade A. (2022). Evaluation of the Formulation Parameter-Dependent Redispersibility of API Nanoparticles from Fluid Bed Granules. Pharmaceutics.

[24] Jakubowska E., Bielejewski M., Milanowski B., Lulek J. (2022). Freeze-drying of drug nanosuspension– study of formulation and processing factors for the optimization and characterization of redispersible cilostazol nanocrystals. J. Drug Deliv. Sci. Technol..

[25] Ma Y., Gao J., Jia W., Liu Y., Zhang L., Yang Q., Guo J., Zhao J., Yan B., Wang Y. (2020). A Comparison of Spray-Drying and Freeze-Drying for the Production of Stable Silybin Nanosuspensions. J. Nanosci. Nanotechnol..

[26] Sahnen F., Kamps J.P., Langer K. (2020). Conversion of indomethacin nanosuspensions into solid dosage forms via fluid bed granulation and compaction. Eur. J. Pharm. Biopharm..

[27] Schönfeld B., Westedt U., Wagner K.G. (2021). Vacuum drum drying—A novel solvent-evaporation based technology to manufacture amorphous solid dispersions in comparison to spray drying and hot melt extrusion. Int. J. Pharm..

[28] Schönfeld B.V., Westedt U., Keller B.L., Wagner K.G. (2022). Transformation of Ritonavir Nanocrystal Suspensions into a Redispersible Drug Product via Vacuum Drum Drying. AAPS PharmSciTech.

[29] Tan E.H., Parmentier J., Low A., Möschwitzer J.P. (2017). Downstream drug product processing of itraconazole nanosuspension: Factors influencing tablet material properties and dissolution of compacted nanosuspension-layered sugar beads. Int. J. Pharm..

[30] Chaubal M.V., Popescu C. (2008). Conversion of Nanosuspensions into Dry Powders by Spray Drying: A Case Study. Pharm. Res..

[31] FDA Venclexta (Venetoclax) Clinical Pharmacology and Biopharmaceutics Review(s) Application Number: 208573Orig1s000. https://www.accessdata.fda.gov/drugsatfda_docs/nda/2016/208573Orig1s000ClinPharmR.pdf.

[32] Souers A.J., Leverson J.D., Boghaert E.R., Ackler S.L., Catron N.D., Chen J., Dayton B.D., Ding H., Enschede S.H., Fairbrother W.J. (2013). ABT-199, a potent and selective BCL-2 inhibitor, achieves antitumor activity while sparing platelets. Nat. Med..

[33] Cang S., Iragavarapu C., Savooji J., Song Y., Liu D. (2015). ABT-199 (venetoclax) and BCL-2 inhibitors in clinical development. J. Hematol. Oncol..

[34] Itchaki G., Brown J.R. (2016). The potential of venetoclax (ABT-199) in chronic lymphocytic leukemia. Ther. Adv. Hematol..

[35] B-290 Data Sheet 11594126 H en 1907.

[36] Zuo B., Sun Y., Li H., Liu X., Zhai Y., Sun J., He Z. (2013). Preparation and in vitro/in vivo evaluation of fenofibrate nanocrystals. Int. J. Pharm..

[37] Ćurić A., Keller B.-L., Reul R., Möschwitzer J., Fricker G. (2015). Development and lyophilization of itraconazole loaded poly(butylcyanoacrylate) nanospheres as a drug delivery system. Eur. J. Pharm. Sci..

[38] Malamatari M., Somavarapu S., Kachrimanis K., Buckton G., Taylor K.M.G. (2017). Preparation of respirable nanoparticle agglomerates of the low melting and ductile drug ibuprofen: Impact of formulation parameters. Powder Technol..

[39] Sun W., Ni R., Zhang X., Li L.C., Mao S. (2015). Spray drying of a poorly water-soluble drug nanosuspension for tablet preparation: Formulation and process optimization with bioavailability evaluation. Drug Dev. Ind. Pharm..

[40] Li J., Zhou Y., Aisha M., Wu J., Wang H., Huang F., Sun M. (2021). Preparation of loratadine nanocrystal tablets to improve the solubility and dissolution for enhanced oral bioavailability. J. Pharm. Pharmacol..

[41] Malamatari M., Charisi A., Malamataris S., Kachrimanis K., Nikolakakis I. (2020). Spray Drying for the Preparation of Nanoparticle-Based Drug Formulations as Dry Powders for Inhalation. Processes.

[42] Torge A., Grützmacher P., Mücklich F., Schneider M. (2017). The influence of mannitol on morphology and disintegration of spray-dried nano-embedded microparticles. Eur. J. Pharm. Sci..

[43] Sun C.C. (2008). Mechanism of moisture induced variations in true density and compaction properties of microcrystalline cellulose. Int. J. Pharm..

[44] Osei-Yeboah F., Sun C.C. (2023). Effect of drug loading and relative humidity on the mechanical properties and tableting performance of Celecoxib–PVP/VA 64 amorphous solid dispersions. Int. J. Pharm..

[45] Ohrem H.L., Schornick E., Kalivoda A., Ognibene R. (2014). Why is mannitol becoming more and more popular as a pharmaceutical excipient in solid dosage forms?. Pharm. Dev. Technol..

[46] Chaudhary R.S., Patel C., Sevak V., Chan M. (2018). Effect of Kollidon VA^®^64 particle size and morphology as directly compressible excipient on tablet compression properties. Drug Dev. Ind. Pharm..

[47] Kolter K., Flick D. (2000). Structure and Dry Binding Activity of Different Polymers, Including Kollidon^®^ VA 64. Drug Dev. Ind. Pharm..

[48] Schmidt P.C., Lang S. (2013). Pharmazeutische Hilfsstoffe: Eigenschaften, Anwendung und Handelsprodukte.

[49] Kosugi A., Leong K.H., Tsuji H., Hayashi Y., Kumada S., Okada K., Onuki Y. (2020). Characterization of Powder- and Tablet Properties of Different Direct Compaction Grades of Mannitol Using a Kohonen Self-organizing Map and a Lasso Regression Model. J. Pharm. Sci..

[50] Skelbæk-Pedersen A.L., Al-Sharabi M., Vilhelmsen T.K., Rantanen J., Zeitler J.A. (2020). Effect of particle size and deformation behaviour on water ingress into tablets. Int. J. Pharm..

[51] Kitazawa S., Johno I., Ito Y., Teramura S., Okado J. (1975). Effects of hardness on the disintegration time and the dissolution rate of uncoated caffeine tablets. J. Pharm. Pharmacol..

[52] Yang Y., Liu J., Hu A., Nie T., Cheng Z., Liu W. (2022). A Critical Review on Engineering of d-Mannitol Crystals: Properties, Applications, and Polymorphic Control. Crystals.

[53] Peltonen L., Strachan C.J. (2020). Degrees of order: A comparison of nanocrystal and amorphous solids for poorly soluble drugs. Int. J. Pharm..

[54] Quodbach J., Kleinebudde P. (2016). A critical review on tablet disintegration. Pharm. Dev. Technol..

[55] Sun C.C. (2017). Microstructure of Tablet—Pharmaceutical Significance, Assessment, and Engineering. Pharm. Res..

