# Supplementary material for: Transformation of ABT-199 Nanocrystal Suspensions into a Redispersible Drug Product—Impact of Vacuum Drum Drying, Spray Drying and Tableting on Re-Nanodispersibility
Source: Pharmaceutics. 2024 Jun 8;16(6):782. doi: 10.3390/pharmaceutics16060782 (PMC11207449; doi:10.3390/pharmaceutics16060782)
Supplement: Supplementary file 1 [file pharmaceutics-16-00782-s001.zip › pharmaceutics-3018560-supplementary.pdf]

pharmaceutics

MDPI

Supplementary Materials:

Transformation of ABT-199 Nanocrystal Suspensions into a Redispersible Drug Product—Impact of Vacuum Drum Drying, Spray Drying and Tableting on Re-Nanodispersibility

Barbara Schönfeld, Julius Sundermann, Benjamin-Luca Keller, Ulrich Westedt * and Oliver Heinzerling

AbbVie Deutschland GmbH & Co. KG, Knollstraße 50, 67061 Ludwigshafen, Germany; barbara.schoenfeld@abbvie.com (B.S.); julius.sundermann@abbvie.com (J.S.); benjamin-luca.keller@abbvie.com (B.-L.K.); oliver.heinzerling@abbvie.com (O.H.)

* Correspondence: ulrich.westedt@abbvie.com

Table S1. Particle size results of ABT-199 nanosuspension sub-batches by laser diffraction.

Batch	D10 [nm]	D50 [nm]	D90 [nm]	Nano < 1µm [%]
#1	27.2	119	567	94.8
#2	24.9	101	433	96.9
#3	24.7	99	417	97.2
Mean	25.6	106	472	96.3
SD	1.4	10.7	82.5	1.3

Table S2. Particle size results of merged ABT-199 nanosuspension using dynamic light scattering and laser diffraction.

	D10 [nm]	D50 [nm]	D90 [nm]	Nano <1µm [%]
1	26.1	111	476	96.5
2	25.8	110	473	96.5
3	26.4	114	480	96.5
4	25.3	106	456	96.7
5	25.3	107	456	96.7
6	25.2	105	453	96.7
Mean	25.7	109	466	96.6
SD	0.5	3.4	11.9	0.1

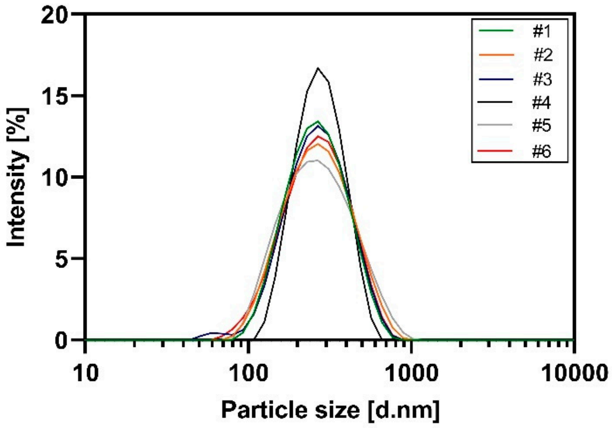

Figure S1. Intensity-based particle size distribution of initial nanosuspension (merged nanosuspension of run #1-3)

Table S3. Results of drying process and intermediate characterization (LOD= loss on drying; FFC=flow function coefficient).

	Formulation	Yield [%]	LOD [%]	Particle density [g/cm³]	Bulk density [g/cm³]	Tapped density [g/cm³]	FFC	Particle size distribution (solid) [nm]		
								D10	D50	D90
VDD	Man_22%DL	89	1.41 ± 0.10	1.3992 ± 0.0011	0.394 ± 0.008	0.505 ± 0.014	12.81 ± 3.76 (free flowing)	49.7 ± 10.8	294.0 ± 44.4	634.3 ± 88.9
	Man_33%DL	93	1.60 ± 0.03	1.3710 ± 0.0005	0.378 ± 0.002	0.473 ± 0.004	10.36 ± 2.32 (free flowing)	61.5 ± 1.6	260.3 ± 14.2	578.0 ± 25.5
	Man_44%DL	94	1.51 ± 0.07	1.3368 ± 0.0006	0.373 ± 0.001	0.491 ± 0.002	12.49 ± 2.15 (free flowing)	58.0 ± 1.5	210.7 ± 10.3	471.7 ± 32.9
	ManTre_22%DL	83	2.15 ± 0.07	1.4057 ± 0.0011	0.377 ± 0.003	0.489 ± 0.012	3.51 ± 0.07 (cohesive)	59.9 ± 3.4	251.0 ± 20.5	578.3 ± 40.5
	ManTre_33%DL	90	1.75 ± 0.08	1.3725 ± 0.0010	0.313 ± 0.006	0.416 ± 0.003	3.75 ± 0.44 (cohesive	40.5 ± 0.8	149.0 ± 4.6	351.0 ± 15.4
	ManTre_44%DL	90	1.58 ± 0.03	1.3312 ± 0.0012	0.306 ± 0.005	0.401 ± 0.001	4.33 ± 0.22 (easy flowing)	39.6 ± 1.1	149.0 ± 9.0	398.7 ± 39.7
	Tre_22%DL	78	2.27 ± 0.13	1.4067 ± 0.0010	0.186 ± 0.001	0.283 ± 0.002	4.01 ± 0.20 (easy flowing)	20.0 ± 0.6	92.5 ± 3.4	256.3 ± 21.2
	Tre_33%DL	79	1.90 ± 0.08	1.3681 ± 0.0011	0.206 ± 0.006	0.300 ± 0.005	4.56 ± 0.05 (easy flowing)	26.5 ± 0.6	112.3 ± 5.5	288.3 ± 10.6
	Tre_44%DL	81	1.73 ± 0.10	1.3279 ± 0.0012	0.252 ± 0.002	0.348 ± 0.003	4.87 ± 0.47 (easy flowing)	33.9 ± 1.0	141.0 ± 4.4	347.3 ± 14.6
SD	Man_22%DL	68	1.48*	1.4044 ± 0.0008	0.322 ± 0.005	0.560 ± 0.005	2.41 ± 0.01(cohesive)	3.5 ± 0.5	18.7 ± 1.4	59.8 ± 5.1
	Man_33%DL	89	1.29*	1.3570 ± 0.0013	0.285 ± 0.011	0.487 ± 0.007	1.98 ± 0.12 (very cohesive.)	2.9 ± 0.2	9.39 ± 0.3	250 ± 221.0
	Man_44%DL	69	1.43*	1.3135 ± 0.0009	0.298 ± 0.008	0.508± 0.013	2.48 ± 0.19(cohesive)	2.6 ± 0.0	8.33 ± 0.3	242 ± 197.0
	ManTre_22%DL	15	3.19*	1.4128 ± 0.0030	0.249 ± 0.009	0.319 ± 0.010	4.73 ± 0.12 (easy flowing)	20.6 ± 5.0	388 ± 54.8	911 ± 28.8
	ManTre_33%DL	27	3.19*	1.3776 ± 0.0023	0.231 ± 0.002	0.333 ± 0.004	2.33 ± 0.03(cohesive)	6.4 ± 0.1	171 ± 3.9	879 ± 67.3
	ManTre_44%DL	40	3.31*	1.3577 ± 0.0025	0.169 ± 0.015	0.235 ± 0.042	1.75 ± 0.1 7(very cohesive)	2.8 ± 0.0	7.21 ± 0.1	627 ± 70.2
	Tre_22%DL	44	4.67*	1.3688 ± 0.0053	0.254 ± 0.008	0.498 ± 0.011	1.91 ± 0.04 (very cohesive)	3.1 ± 0.1	11.3 ± 0.3	192 ± 57.0
	Tre_33%DL	46	2.73*	1.3750 ± 0.0031	0.201 ± 0.005	0.350 ± 0.012	1.89 ± 0.03 (very cohesive)	2.7 ± 0.0	8.56 ± 0.1	640 ± 81.9
	Tre_44%DL	65	3.16*	1.3469 ± 0.0011	0.219 ± 0.001	0.366 ± 0.002	1.67 ± 0.03 (very cohesive)	2.2 ± 0.1	5.75 ± 0.2	44.7 ± 49.5

*= measured as n=1 due to low yields

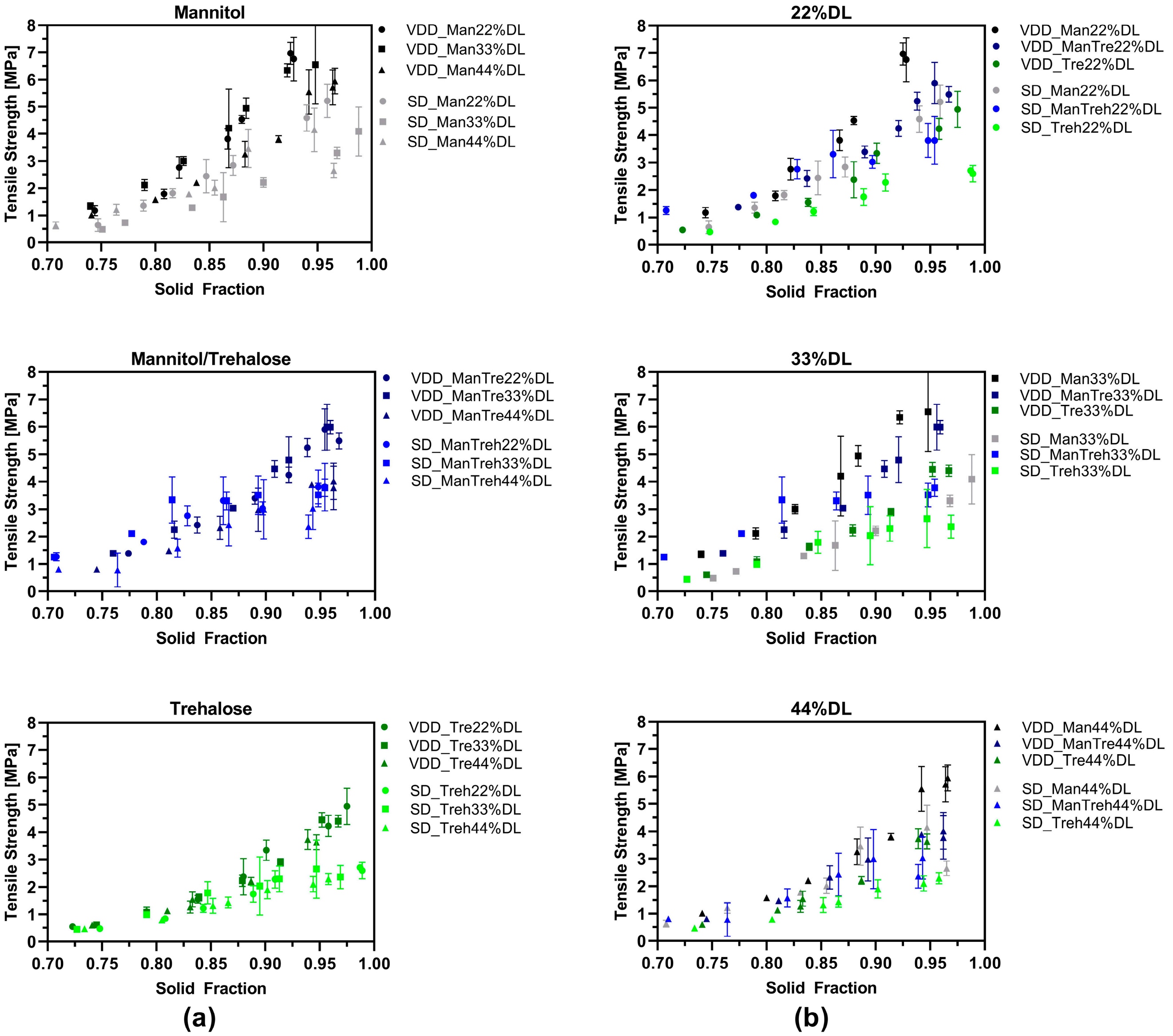

Figure S2. Compactability - simulating KorschXL100 at 30 rpm turret speed: (a) grouped by drying protectant used comparing VDD and SD formulations at different ABT-199 drug loads; (b) grouped by ABT-199 drug loads comparing VDD and SD formulations containing different drying protectants

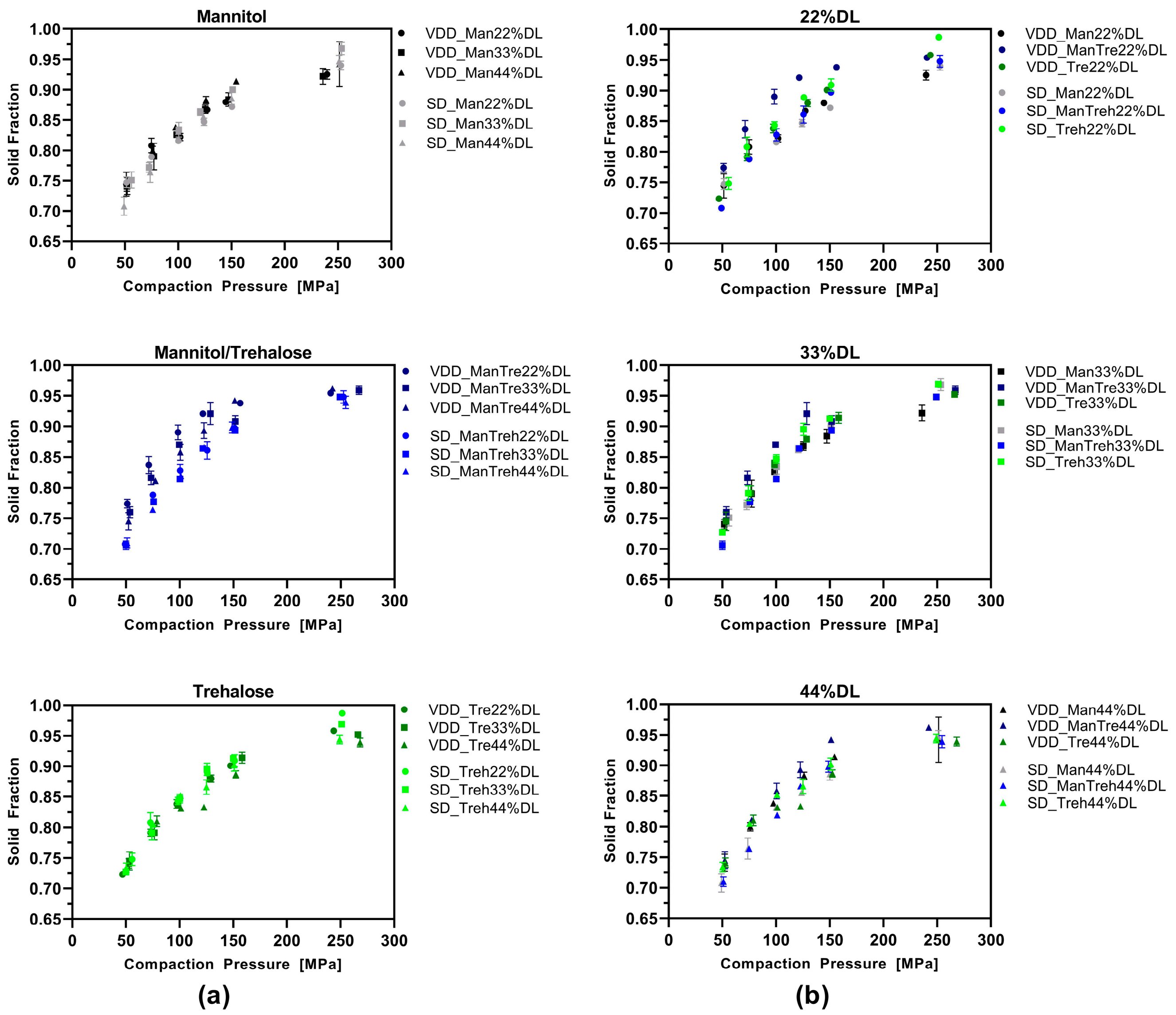

Figure S3. Compressibility - simulating KorschXL100 at 30 rpm turret speed: (a) grouped by drying protectant used comparing VDD and SD formulations at different ABT-199 drug loads; (b) grouped by ABT-199 drug loads comparing VDD and SD formulations containing different drying protectants

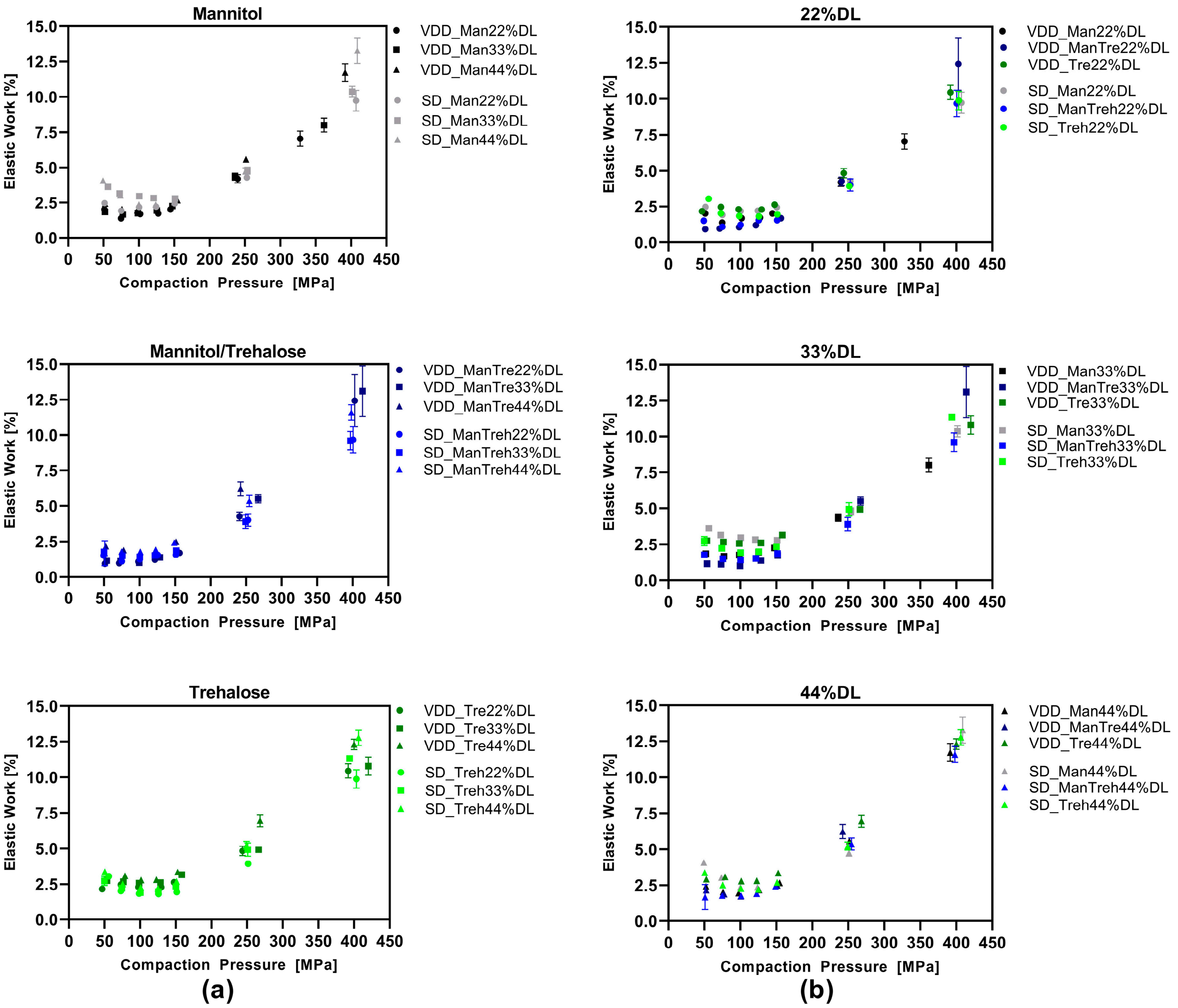

Figure S4. Elastic Work - simulating KorschXL100 at 30 rpm turret speed: (a) grouped by drying protectant used comparing VDD and SD formulations at different ABT-199 drug loads; (b) grouped by ABT-199 drug loads comparing VDD and SD formulations containing different drying protectants
